# Effect of Multimedia Self-Care Education on Quality of Life in Burn Patients

**Published:** 2017-09

**Authors:** Fatemeh Mohaddes Ardebili, Tahereh Najafi Ghezeljeh, Mehri Bozorgnejad, Mohammadreza Zarei, Hooman Ghorbani, Farzad Manafi

**Affiliations:** 1 Department of Medical-Surgical Nursing, Faculty of Nursing and Midwifery, Iran University of Medical Sciences, Tehran, Iran; 2 Department of Critical Care Nursing, School of Nursing and Midwifery, Iran University of Medical Sciences, Tehran, Iran; 3 Motahari Burn Hospital, Iran University of Medical Sciences, Tehran, Iran; 4 School of Medicine, Iran University of Medical Sciences, Tehran, Iran

**Keywords:** Education, Multimedia self-care program, Quality of life, Burn injury

## Abstract

**BACKGROUND:**

Burn injuries can have adverse effects on quality of life of patients and can disturb their physiological, psychological, social and spiritual well-being. This study aimed to investigate the effect of multimedia self-care program on quality of life in burn patients.

**METHODS:**

This Randomized controlled clinical trial was conducted from November 2015 to December 2016. The samples were hospitalized burn patients with 10% to 45% of 1^st^, 2^nd^ and 3^rd^ degree burns of total body surface area (TBSA). The patients were randomly allocated into experimental (n=50) and control (n=50) groups. Both groups received the routine in-person self-care trainings of the hospital and then the experimental group received self-care compact disks. The quality of life questionnaire in both groups were completed before intervention and after 3-months and statistically analyzed.

**RESULTS:**

Accordingly, the changes in quality of life and the dimensions between both groups after 3 month of intervention were significant. The changes in quality of life in experiment group was significantly greater than control group for physical, psychological and social variables.

**CONCLUSION:**

According to the findings, using multimedia self-care programs can improve burn patient’s quality of life, so it is recommended for nurses and hospital staffs of burn injury wards to use multimedia self-care programs as a complementary therapy measure.

## INTRODUCTION

Burn injuries having unpleasant physical, economic, social, psychological and emotional consequences which seriously endanger the health and quality of life.^1^ Nowadays, in addition to considering the death rate index, it should be noted that health and hygienic dimensions in which quality of life is more important because of lots of physical, psychological, social, and economic complications occurring after discharge, such as skin problems, scarring, pain, itching, stress, low self-esteem, anxiety, depression, posttraumatic stress disorder, therefore the quality of life of these patients is reduced after the burn injuries. Quality of life in adult is based on happiness and satisfaction which influence on the well-being of people with physical, mental, social and role functioning and communicational performance.^2^^-^^4^

One of the major goals of health care systems is to maximize the performance and to improve the quality of life in the daily life. In this processes, the patients’ self-care strategies improve the self-management and reduces the pain and other disorders. However, the patient will acquire enough knowledge and skill to make decisions and solve the self-related problems.^5^ Nurses, as an important health care member having a crucial role in caring and increasing self-care health knowledge, rehabilitation and improving quality of life of patients. Through possible intervention, nurses have the ability to direct their patients’ life to balance and affect their quality of life.^6^

Patient’s self-care education helps to increase health care programs and to decrease relapse rates and frequent clinic attending. Lack of self-care knowledge is one of the most important reasons for patient’s rehospitalization.^7^ In multimedia self-care programs, contents are offered through, at least, two elements such as text, voice, image, video or animations. Moreover, they are accessible any time.^8^ Multimedia self-care programs, as a methodology can facilitate patient’s self-care learning.^9^ Since there have been few studies regarding self-care education and quality of life of burn patients, and with regard to significance of patients’ rehabilitation to self-care for their quality of life, researchers tried to conduct this study on the effect of multimedia self-care education on the quality of life in burn patients. 

## MATERIALS AND METHODS

A randomized clinical trial study was conducted on 100 eligible hospitalized burn patients from both genders who were selected using easy sampling method. The patients were allocated randomly into two groups of experimental and control. The samples were10% to 45% burn of total body surface area (TBSA) with degrees of 2^nd^ and 3^rd^, or sets of 1^st^, 2^nd^, and 3^rd^ who were accessible. With regard to the previous studies on quality of life and effect of self-care education, the number of samples were 50 in each group, with confidence interval of 95%, test power of 80%, and the minimum acceptable differences of 10 in quality of life for both groups with10% drop-out rate, totally including 100 sample size. 

Before the intervention, the informed consent form was signed by all participants of the study, the demographic questionnaire was completed for both experimental and control groups. Then, they received self-care routine recommendations face to face. The experimental group received a multimedia self-care CD (The contents were designed for burn patients, CD-based educational books and educational resources).^5^ The contents were also approved by educational experts in Motahari Burn Injury Hospital in Tehran. The self-care included movement and activities, daily works and social relations, care of repaired burn areas and areas of grafted skin, nutrition, compression clothing or garment recommendation for mental health care, sleep improvement, and pharmacological care. Moreover, they also received an in-person briefing session for possible questions for the Cd at patients discharge.

Questionnaire of patient’s quality of life (Brief burn specific health scale) once before intervention at patients’ discharge and 3 months after intervention were completed by the subjects of both groups. To complete the questionnaire, it is worth mentioning that the forms were completed via phone call. The questionnaire of quality of life of burn patients (the Burn specific health scale: BSH-B) was designed and used in 2001^10^ and in Iran in 2010, 2013 and 2014.^11^^-^^13^ In this study, the BSH-B content validity was reviewed and corrected by 10 university faculty members of medical universities. The reliability was measured through completion of the questionnaire by 20 burn patients admitted in Motahari Hospital and repeating it after 15 days determined by test-retest, Cronbach alpha coefficient of 0.89.

The questionnaire included 40 questions (18 questions were related to physical, 11 to mental and 11 about social & quality of life aspects) about how to care the burn site, the job, communication, the ability to do simple activities, and also their textual performance. They also had options of high, low, medium, extremely and never which were scored from 1 to 5, respectively. Therefore, each question had a minimum score of one and maximum point of 5. 

To analyze the data, descriptive and deductive statistics was used. To study distributive homogeneity of demographic features in both experimental and control groups, chi-squared and Exact Fisher’s tests were applied. To assess the normality of distribution of data, Kolmogorov-Smirnov test was used. To compare the quality of life among both groups, nonparametric sign test of Mann Whitney was applied. To compare the mean scores of quality of life before and after 3 months of intervention in both groups, Mann Whitney test was used. Statistical analysis was undertaken by SPSS software (Version 20, Chicago, IL, USA). The ethical approval of this study was also granted from Research Ethical Committee of Iran Medical Sciences University

## RESULTS

In this study, there were totally 100 patients including males and females. In experimental group, 34.90% of the subjects were between 39-48 years old, and in control group; 44.90% of them were between 29-38 years. The main cause of burn injuries was flame (35%), the deepest burn (62%) among both groups were 1^st^, 2^nd^, and 3^rd^ degree of burns. The TBSA among most individuals was between 21% and 26% in both groups. Regarding occupation and marital status, there was a significant statistical difference between groups; but the result of one-way ANOVA analysis showed no significant statistical differences for quality of life between groups, so these two variables were not intervening variables (Table 1). 

**Table 1:** Relative and Absolute frequency distribution of demographic features of both groups of experimental and control

**Frequency variables**		**Experiment**		**Control**		**P value**
		**No.**	**%**	**No.**	**%**	
Gender	Female	28	56.00	22	56.00	0.23
	Male	23	44.00	28	44.00	
Age	28-18	11	22.00	10	20.40	0.35
	38-29	15	30.00	22	44.90	
	48-39	17	34.00	14	28.60	
	58-49	7	14.00	6	6.10	
Marital status	single	28	56	10	20.40	0.001
	Married	22	44	39	79.60	
	Divorced	0	0	0	0	
Occupation	Employed	24	48.00	30	62.50	0.006
	Housewife	12	24.00	16	33.30	
	Jobless	14	28.00	4	4.20	
Level of education	Under diploma	2	4.20	4	8.90	0.079
	Diploma	26	52.10	33	66.70	
	Bachelor	22	43.80	12	22.20	
	Masters or higher	0	0	1	2.20	
Cause of burn injury	Gas	3	6.00	2	4.00	0.64
	Natural gas	8	16.00	14	28.00	
	Flame	18	36.00	17	34.00	
	Liquids	13	26.00	12	24.00	
	Kerosine	1	2.00	2	4.00	
	Food	4	8.00	1	2.00	
	Etc.	3	6.00	2	4.00	
Percentage of burn	Percent	12	24.00	10	20.00	0.239
	21-26	12	24.00	18	36.00	
	27-32	10	20.00	9	18.00	
	33-38	8	16.00	2	4.00	
	More	8	16.00	11	22.00	
Degree	Degree	30	60.00	32	64.00	0.17
	2 , 3	20	40.00	18	36.00	
Area	Hands, legs	0	0	3	6.30	0.059
	Body, hands, legs	23	46.00	13	25.00	
	Head, shoulder, hands, legs	10	20.00	11	20.80	
	Whole body	17	34.00	23	47.90	

The results in Table 2 show that for total score of quality of life and mental and social dimensions in experiment and control groups, there was statistically a significant difference before the intervention. For physical performance, there was not any significant difference between the groups, so both groups were almost identical. In general, the mean score of quality of life in experimental group was greater than the control group. Three months after the intervention, for mean score of quality of life and the dimension among both groups, the difference was statistically significant and the mean score of quality of life in experimental group was greater than the control group.

**Table 2:** Quality of life score comparison before and after intervention in control and experimental groups.

**Quality of life**	**Group**	**Before**		**After**		**P value**
		**Mean**	**SD**	**Mean**	**SD**	
Mental	Experimental	2.08	0.59	3.37	0.93	0.001
	Control	1.64	0.47	2.24	0.4	0.001
	P value	0.028	0.028		0.001	0.001
Social	Experimental	1.92	0.60	3.29	0.95	0.001
	Control	1.55	0.46	2.15	0.30	0.001
	P value	0.162			0.001	0.001
Physical	Experimental	1.61	0.71	3.44	0.95	0.001
	Control	1.45	0.47	2.32	0.37	
	P value	0.41			0.001	
Total	Experimental	1.87	0.6	3.37	0.93	
	Control	1.55	0.44	2.24	0.37	
	P value		0.162		0.001	

The results in Table 2 show that the increase of quality of life, 3 months after the intervention of multimedia self-care program was effective. In control group before and after entering the study, for quality of life regarding the dimensions, the difference was statistically significant showing an increase in quality of life in the control group after 3 months of entering the study. 

According to the results of Table 3, the changes of the total scores of quality of life and the dimensions in experimental and control groups, the difference was statistically significant. The mean changes of scores of quality of life and the dimension in experimental group was greater than the control group. It should also be noted that the mean score of physical variable in experimental group was 3.44, while in control group was 2.32 showing an increase in physical performance of experimental group after 3 months of intervention as they were homogenous before intervention. 

**Table 3:** Comparison of changes for mean scores of quality of life in experimental and control groups

**Quality of life**	**Group**	**Changes**		
		**Mean**	**SD**	**P value**
Mental	experimental	1.29	0.01	0.01
	Control	0.61	0.51	
Social	experimental	1.37	0.001	0.001
	Control	0.59	0.53	
Physical	experimental	1.83	0.001	0.001
	Control	0.86	0.55	
Total	Experimental	1.59	0.001	0.001
	Control	0.68	0.51	

## DISCUSSION

The outcome of quality of life before and after intervention during the 3-month interval using multimedia self-care program showed that it was effective for an increase in quality of life specially for physical performance. The result of this study corresponds with experimental clinical trial of Hashemi and Colleagues (2014)^11^ aiming to study the effect of Orem’s self-care programs on burn patient’s quality of life. The results showed that the quality of life in experimental group in the first month increased from 73.3% to 83.78% and in the second month to 98.12%, while in the control group no change in quality of life was noted.

Moreover, the result of randomized clinical trial with control group by another study^14^ under the title of the effect of multimedia educational program on knowledge and anxiety, and matching with anti-scar pressure garment in burn patients corresponded with our study results. The findings showed that multimedia education to increase knowledge and to decrease anxiety and dealing with anti-scar garment in comparison to the control group was effective. The result of experimental clinical trial in Australia showed that use of self-care DVDs in comparison to the control group helped to improve self-care of burn patients, corresponded with the present study.^15^

Also Golchin and colleagues studied under the title of the study of the effects of self-care education on quality of life of leukemia patients after 4-month intervention and showed that education helped to improve quality of life.^16^ Also, Hamidizadeh *et al.* in their experimental trial showed the effect of Oren’s self-care educational program on improving physical performance of the quality of life formultiple Sclerosis (MS) patients referred to Iran MS Association after 4 months. In our study, the highest effect of education was for physical performance dimension of patients, too.^17^

In the study of Heidari *et al.*, in Borojen City in Iran, under the title of the study of effect of self-care education program on the quality of elderly showed that a 3-month program increased the quality of life especially for social performance area.^18^ So based on disabilities of burn among both genders and all age groups in developing and developed countries,^19^^,^^20^ the effect of education on improving dimension of quality of life in chronic disease patients is of great importance. So as the multimedia self-care chronic disease patients, multimedia self-care programs which are easily accessible at any time and everywhere, it is recommended to improve the quality of life in burn patients. 
